# Cerebral and occipito-atlanto-axial involvement in mucopolysaccharidosis patients: clinical, radiological, and neurosurgical features

**DOI:** 10.1186/s13052-018-0558-x

**Published:** 2018-11-16

**Authors:** Carlo Giussani, Lelio Guida, Francesco Canonico, Erik P. Sganzerla

**Affiliations:** 1 Department of Neurosurgery, University of Milan-Bicocca, San Gerardo Hospital, via G.B. Pergolesi 33, 20900 Monza, Italy; 2 Department of Neuroradiology, University of Milan-Bicocca, San Gerardo Hospital, Monza, Italy

**Keywords:** Mucopolysaccharidosis, Craniovertebral junction instability, Hydrocephalus, Cerebral atrophy

## Abstract

**Background:**

Neurosurgical features of mucopolysaccharidosis (MPS) patients mainly involve the presence of cranio-vertebral junction (CVJ) abnormalities and the development of communicating hydrocephalus. CVJ pathology is a critical aspect that severely influences the morbidity and mortality of MPS patients. Hydrocephalus is slowly progressing; it must be differentiated from cerebral atrophy, and rarely requires treatment. The aim of this paper was to review the literature concerning these conditions, highlighting their clinical, radiological, and surgical aspects to provide a practical point of view for clinicians.

**Results:**

CVJ involvement may present with cervical pain, unsteady gait, frequent falls, and progressive impairment of autonomous ambulation, an acute tetraplegia even after minor trauma. Magnetic resonance imaging (MRI) of the cervical spine, including active dynamic flexion and extension scans, is the most powerful imaging technique for detecting spinal cord compression at the CVJ in MPS patients. The main radiological features include atlanto-axial subluxation, odontoid hypoplasia, periodontoid soft tissue masses, spinal canal narrowing, and spinal cord compression. Together with MRI, fine-cut computed tomography (CT) scans with coronal and sagittal three-dimensional reconstructions are important diagnostic tools in the preoperative workup thanks to the information gleaned about bone structure conformation and angles. Finally, angio-CT slices are equally useful in preoperative planning, defining vertebral artery position in relation to bony structures. Surgery of the CVJ is proposed both to treat cord compression with MRI signs of myelopathy or as a preventive treatment in patients at high risk of cord damage. Among different surgical options, we always suggest performing decompression and instrumented stabilization.

Hydrocephalus may occasionally present clinically with intracranial hypertension symptoms such as headache, vomiting, and high sight impairment. Neurocognitive symptoms may be hidden by the constitutive cognitive impairment. MRI with a study of dynamic cerebrospinal fluid (CSF) flow is helpful to differentiate from ventriculomegaly, which does not require treatment. Ventriculo-peritoneal shunt placement is the gold standard to treat hydrocephalus, although endoscopic third ventriculostomy has recently shown good results in some patients.

**Conclusion:**

Early recognition of CVJ pathology and hydrocephalus is critical to avoid the development of severe complications. A multidisciplinary approach involving physicians, neuroradiologists, and neurosurgeons is needed to detect such conditions and to select patients eligible for surgery.

## Background

Mucopolysaccharidoses (MPS) are a group of inherited autosomal recessive lysosomal storage diseases (except for MPS II, which is sex-linked) caused by the deficiency of the enzymes involved in the degradation of glycosaminoglycans (GAGs), a major component of connective tissue [1].

Among the several systemic conditions associated with MPS, two major features are of neurosurgical interest: the presence of cranio-vertebral junction (CVJ) abnormalities, especially in Morquio’s disease (MPS IV) [2], and the development of communicating hydrocephalus [3], the occurrence of which differs according to the pathology subtype (Table 1). The former condition is potentially life threatening or progressively invalidating [4]. In fact, abnormalities of the CVJ with spinal stenosis need to be recognized early since they may result in slow and progressive myelopathy or sudden post-traumatic tetraplegia and respiratory failure [5, 6]. On the other hand, hydrocephalus is a slowly progressive condition [3]; it has to be differentiated from cerebral atrophy, and rarely requires treatment. The aim of the present paper is to critically review the clinical, radiological, and surgical management of MPS patients, focusing on both the aforementioned conditions to provide a practical point of view for the clinicians who take care and routinely face these patients and their families.

**Table 1:** The occurrence rate of hydrocephalus/ventriculomegaly, brain atrophy, and spinal stenosis in patients with mucopolysaccharidosis (MPS)

	Hydrocephalus/ventriculomegaly	Atrophy	Spinal stenosis
MPS I			
Hurler	+++	++	++
Hurler/Scheie	++	+	++
Scheie	+++	+	++
MPS II (Hunter)	+++	+++	+
MPS III (Sanfilippo)			
III A	++	+	+
III B	++	+++	–
III C	–	–	–
III D	+	+	–
MPS IV (Morquio)			
IV A	+/−	–	+++
IV B	+/−	–	+++
MPS VI (Maroteaux-Lamy)	+	+	+++
MPS VII	++	–	+
MPS IX	–	–	–

Cerebral pathology is more common in MPS I, II, and III, while spinal involvement principally occurs in MPS IV and VI

Modified from Zareiriou et al. [3]

## Myelopathy in MPS: How to recognize it?

Patients with MPS may present with complex neurological findings due to multiple and diffuse involvement of dural, ligamentous, and bony structures of the cervical, thoracic, and lumbar regions. Moreover, the orthopedic comorbidities that usually coexist in these patients can negatively affect the clinical evaluation both in the interpretation of patient history as well as during the neurologic examination. In our experience, the attention of the examiner should be mainly focused on cervical pain or torticollis, suggestive of CVJ instability (even if very rare), progressive impairment of autonomous ambulation, postural instability, and fatigue and progressive weakening of upper limbs. However, clinical assessment and neuroradiological studies may not localize the exact level of the canal lesion. At this point, electrophysiological studies could be considered for determining the priority of affected levels before myelopathy worsens and becomes irreversible. Electrophysiological studies are also an objective instrument to observe postoperative improvement of neurological symptoms and signs. However, there is uncertainty in the literature about the possible interindividual feasibility of neurophysiological studies because of the anatomical peculiarity of these patients (e.g., multiple spinal curves). Thus, the integration of medical history, the knowledge of the natural course of the disease, neurological examination, and neuroradiology are critical to detect patients eligible for spinal surgery.

## What are the radiological examinations needed to evaluate CVJ myelopathy?

Unrecognized upper cervical cord compression, aggravated by physiological movements of the occipito-cervical and atlanto-axial joints, may result in slow and progressive myelopathy or sudden, unexpected post-traumatic tetraplegia and respiratory failure [5, 6]. Therefore, it is mandatory to regularly assess the morphoanatomical integrity of the cervical spine at regular scheduled intervals. Charrow et al. recently reviewed routine examinations in MPS IV patients, suggesting a complete neurological evaluation and magnetic resonance imaging (MRI) scan every year [7]. MRI, including active dynamic flexion and extension scans, is the most powerful imaging technique to detect spinal cord compression at the CVJ level in MPS patients [3, 8]. It clearly documents the presence of spinal canal narrowing, signs of spinal cord compression, and spinal cord signal alteration. Together with the routinely performed T1- and T2-weighted axial and sagittal MRI scans, steady-state free-procession (SSFP) sequences provide interesting images that can be used to study the presence of subarachnoid cerebrospinal fluid (CSF) in the narrowed upper cervical canal [9], thus helping in the recognition of stenosis severity through the Space Available for the Cord (SAC) scale [8].

A fine-cut computed tomography (CT) scan with coronal and sagittal three-dimensional reconstructions is a powerful instrument for examining the CVJ. Nevertheless, CT studies of the CVJ should be reserved for children whose MRI has evidenced significant pathological features leading to consideration of a surgical procedure [7].

Atlanto-axial (C1/C2 subluxation) and occipito-atlanto-axial instability, diagnosed by the Atlanto-Dental Interval (ADI) > 5 mm [10] and dynamic sequences, is seldom observed in MPS [4, 11]. Major radiological features to be considered in MPS include odontoid hypoplasia, periodontoid soft tissue masses (due to GAG accumulation behind the odontoid process), and cervical canal stenosis linked to fibrocartilage reactive hypertrophy associated with hypertrophy of the dura and ligamentum flavum [3], with or without MRI signs of cervical myelopathy (see Fig. 2 below).

In addition to cervical stenosis, multilevel subaxial stenosis has been reported, especially in MPS VI (Maroteaux-Lamy), mainly due to posteriorly protruding intervertebral discs, thickened dura, and hypertrophy of the ligamentum flavum [12].

In a previous study, we reviewed radiological features in a consecutive series of 42 MPS patients followed at our Center for Metabolic Diseases, including 12 MPS I, 15 MPS II, 2 MPS III, 9 MPS IV, and 4 MPS VI patients. CVJ abnormalities were frequent in the whole series of MPS patients, with a reduced diameter of the spinal canal at the CVJ in 40% of patients. The most severe spinal canal stenosis and cord compression were observed in MPS IV (33%) and MPS VI (50%). MRI signs of myelopathy were present in only 7% of cases, all affected by MPS IV, highlighting the importance of a regular scheduled diagnostic follow-up in these patients. Increased canal stenosis during dynamic MRI studies in flexion and extension was seen in 67% of MPS IV patients. Dens hypoplasia was present in 79% of cases and was a constant feature in MPS IV and VI. However, due to the low mean age of the children in the series, it was difficult in some cases to differentiate dens hypoplasia from a “physiological” incomplete development of C2 dens [13].

## When to perform surgery in CVJ pathology?

Clinical and/or radiological evidence of acute or progressive myelopathy due to spinal cord compression at the CVJ represents an absolute indication for surgery since the natural history of untreated patients is ineluctably progressing to major neurological deficits [14, 15].

In asymptomatic patients without evidence of myelopathy, preventive treatment is still a matter of debate. It would be recommended in all patients affected by MPS IV (Morquio) with MRI evidence of stenosis and instability at the CVJ, with canal narrowing > 50% [11]. In our experience, surgery is indicated in cases of severe spinal canal narrowing and complete obliteration of perimedullary subarachnoid spaces (SAC < 1 mm), reduction of the upper cervical canal width of more than 50%, and MRI signs of instability (spinal canal stenosis and cord compression) [4, 13]. Whenever possible, in very young patients we suggest delaying the time of surgery until 3 years to obtain sufficient bone maturation, although surgery can be successfully performed even at a younger age as demonstrated in a 17-month-old symptomatic boy by Dickerman et al. [15].

## How to perform surgery in CVJ pathology?

As already underlined, CT axial and sagittal scans are essential in preoperative planning to exactly assess bone geometry and diameters and thus to help choose the most appropriate hardware placement in tailored surgical procedures with screwing techniques. In selected cases, angio-CT delineating the course of vertebral arteries may be indicated, especially if the suitability of C1/C2 transarticular screws is evaluated [7].

Thanks to the improvement in preoperative radiological workup allowing the identification of individual anatomical bone features, the development of several techniques and hardware for instrumented bone fusion, and the improvement in anesthesiological techniques and tools, CVJ surgery has become safer and more feasible with an acceptable complication rate if the associated medical problems and anesthesiological challenges are recognized and managed appropriately. Surgical options include decompression of the spinal canal or decompression associated with spinal stabilization. In MPS other than Morquio disease without radiological evidence of occipito-atlanto-axial instability, simple posterior decompression has been advocated as a valid treatment option. In our experience, and since all MPS patients have a constitutive connective tissue weakness even in the absence of a suggestive radiological pattern, we recommend decompression and fusion in most cases. Only in selected adult patients with fully ossified dens and no radiological evidence of instability can a simple decompression be considered. In fact, posterior decompression alone needs the removal of the C1 posterior ring, of the thickened ligamentum flavum, and of the occipito-atlantal membrane, actions that can worsen CVJ instability. This is also almost constantly present in MPS patients because of odontoid dysplasia with a partially cartilaginous dens, incomplete ossification of anterior and/or posterior arches of C1, and ligamentous laxity [11]. The worsening of spinal instability may expose patients to acute post-traumatic myelopathy, even following a minor trauma in flexion, as shown in a 6-year-old child affected by MPS IV in our series (see Case 1 below).

Recent advances in fixation technologies have enhanced our ability to achieve stable posterior cervical fusions with a variety of screwing techniques which are adaptable to the individual anatomical features of MPS patients, and they should be preferred for their superior biomechanical stability. Among the most widespread surgical options, C1/C2 transarticular screws, C1 lateral mass screws, C1 Olerud clamps, C2 pars screws, and C2 translaminar screws, or a combination of them as anchors for occipito-cervical U-loop constructs can be taken into consideration [16–18]. The choice should be based on fine-cut three-dimensional reconstructed preoperative CT studies. In addition to this hardware, autologous bone grafts from the iliac crest, rib graft, or calvarial bone will be used to increase posterior fixation and achieve biological bone fusions. The development of these technique has progressively superseded the use of external halo orthoses and posterior wiring techniques. Nevertheless, wiring techniques can still be considered in selected patients, achieving good clinical results and long-term CVJ fusion [18]. Traditionally, an irreducible anterior compression deserves transoral microsurgical or video-assisted/transnasal video-assisted anterior decompression and posterior fusion double or one-stage combined in childhood [19]. Nevertheless, CVJ instrumentation and fusion procedures are also indicated in cases not apparently reducing both preoperatively and intraoperatively. In fact, a reduction during the instrumentation can be obtained due to a supposed lever effect produced by the hardware itself, under curarization and myorelaxation; thus a preliminary posterior fixation can always be suggested. Such a philosophy has been named “the always posterior strategy” [20].

## How to differentiate ventricular dilatation due to cerebral atrophy from communicating hydrocephalus: clinical and radiological features

To avoid unneeded ventriculo-peritoneal shunt (VPS) procedures, and consequently the burden of this surgery and its complications, it is of paramount importance to differentiate the frequent condition of ex-vacuo ventricular dilatation (not susceptible to neurosurgical intervention) from a true communicating hydrocephalus [3, 8]. Ventricular enlargement is a common feature in MPS, especially MPS I and II, with most of these patients showing ventriculomegaly. This is believed to be the result of cerebral atrophy secondary to neuronal death and gliosis induced by the accumulation of GAGs, venous hypertension, and defective CSF reabsorption. Instead, hydrocephalus would be the result of GAG deposition in the meninges, impairing the function of pacchionian granulations and causing a communicating hydrocephalus [21]. Differentiation between communicating hydrocephalus and brain atrophy is a demanding task because both conditions share common neuroradiologic features (enlarged ventricular and subarachnoid spaces). In MRI studies, the presence of dilated brain sulci (either diffuse or focal) would suggest brain atrophy and, consequently, ventriculomegaly [22]. Moreover, due to the clinical and radiological similarity of MPS hydrocephalus and adult idiopathic normal pressure hydrocephalus, CSF dynamic sequences (in particular, reversed aqueductal cerebrospinal fluid net flow) may be suggested as an integrative diagnostic tool to evaluate the disruption of CSF circulation leading to hydrocephalus [23]. Despite these advanced techniques, neuroimaging is not always invalidating in differential diagnosis. Therefore, in selected patients the use of invasive techniques such as lumbar puncture, tap-test, CSF pressure monitoring tests, as well as the positioning of intraparechimal pressure catheter, may be necessary to appropriately select patients for surgery. Finally, fundoscopy and visual evoked potentials are not recognized as useful tools to differentiate these two conditions since chronic papilledema in the absence of intracranial hypertension is a common feature of MPS patients (especially Hunter), probably due to GAG deposition within the sclera [24].

## How to treat hydrocephalus in MPS patients?

When a real hydrocephalus is diagnosed, surgical options include VPS and endoscopic third ventriculostomy (ETV). The latter consists of an endoscopically performed stoma at the level of the third ventricle floor so that CSF is shunted directly to basal cisterns, thus bypassing the infratentorial components of the ventricular system. This technique has been primarily advocated for patients with obstructive hydrocephalus, especially due to aqueductal stenosis. There is also growing interest for ETV in MPS patients, as in the case described by Da Silva Neto et al. with clinical and radiological improvement [25]. Technically, the procedure is demanding because of the anatomical peculiarities of the ventricular system, with the major prominence of thalami and the thickness of third ventricle floor infiltrated by undegraded GAG. However, VPS placement remains the most used and successful technique to treat hydrocephalus in MPS patients, considering the etiology (communicating hydrocephalus) and the progressive evolution of this disease. As highlighted by Aliabadi et al., VPS is an effective treatment, especially before stem cell transplantation, and it appears that pretransplantation shunting allows better outcomes [26].

## Case studies

### Case 1: Acute post-traumatic myelopathy

**Fig. 1:**
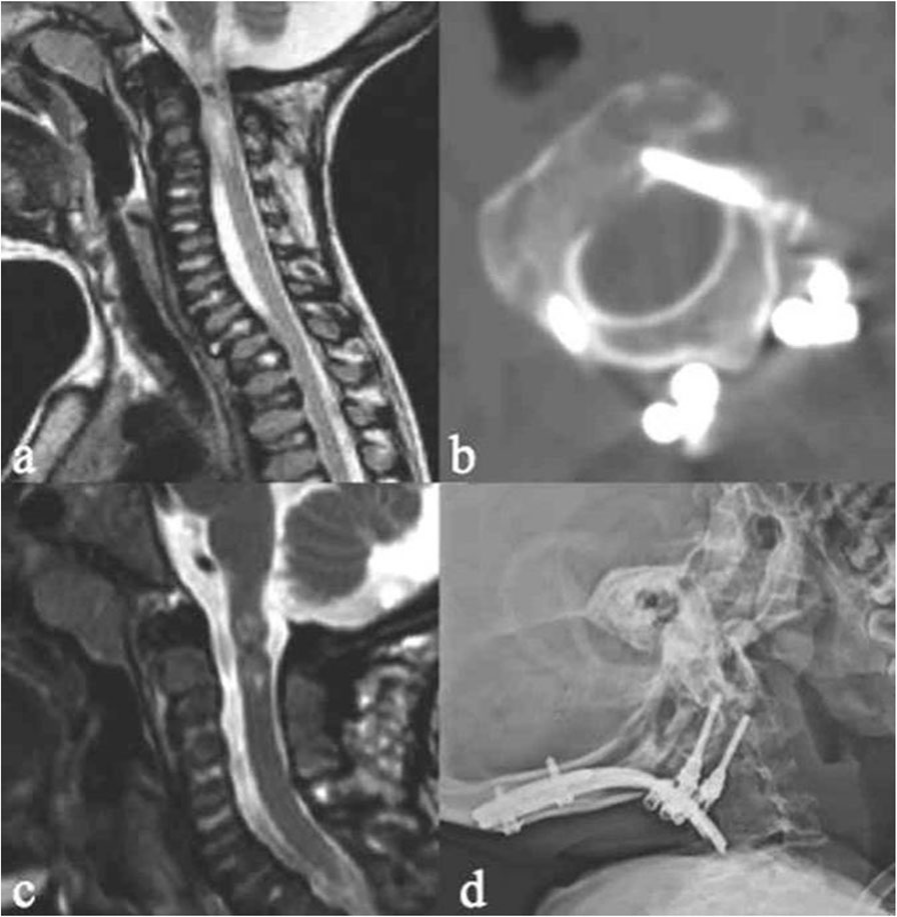
**a** MRI showing severe post-traumatic myelopathy after a minor backwards fall in a previously simply decompressed MPS IV child. **b** C2 pars screws, **c** postoperative MRI, and **d** plain x-rays at 4 years follow-up

A 6-year-old girl with MPS IVA who had previously undergone CVJ decompression at another institution for severe canal stenosis and mild myelopathy, with removal of the posterior arch of C1 and of the thickened atlanto-occipital membrane and ligamentum flavum. After a backwards fall from a child’s chair, she developed acute quadriplegia with respiratory failure (Ranawat IIIB) and was admitted to our neurological intensive care unit. MRI showed an impressive alteration of spinal cord signal at C0–C1 (Fig. 1a). This patient was initially stabilized with an external halo orthosis and submitted to inpatient rehabilitation for some weeks afterwards. After an initial neurological improvement and cardiorespiratory stability, she underwent internal stabilization with C2 pars screws (Fig. 1b) anchored to an occipito-cervical U-loop and occipito-C2 calvarial bone graft. At the 4-year follow-up examination she was able to walk with crutches (Ranawat IIIA). Radiological follow-up examinations revealed wide canal decompression and a stable construct (Fig. 1c, d). This case supports the evidence that stabilization should be always recommended and that the placement of an external orthosis may still represent a valid treatment option in selected cases (e.g., impossibility to perform surgical intervention for respiratory instability).

### Case 2: Progressive myelopathy

A 6-year-old male with MPS VI. After a minor fall he experienced a transient tetraparesis with quick recovery of ambulation. In the following months he suffered from recurrent urinary tract infections. Cervical MRI documented severe stenosis and cord compression at the CVJ with spinal cord signal alterations. Physical examination evidenced pyramidal signs and a urodynamic study was diagnostic for neurological bladder. A posterior cervical decompression and stabilization with C2 pars screws anchored to an occipito-cervical U-loop and calvarial bone graft was then performed. During the follow up, there was a slow recovery of bladder function and normal daily activities. Radiological follow-up examination revealed good canal decompression, stable construct, and steady neurological conditions.

### Case 3: Preventive surgery in CVJ instability

**Fig. 2:**
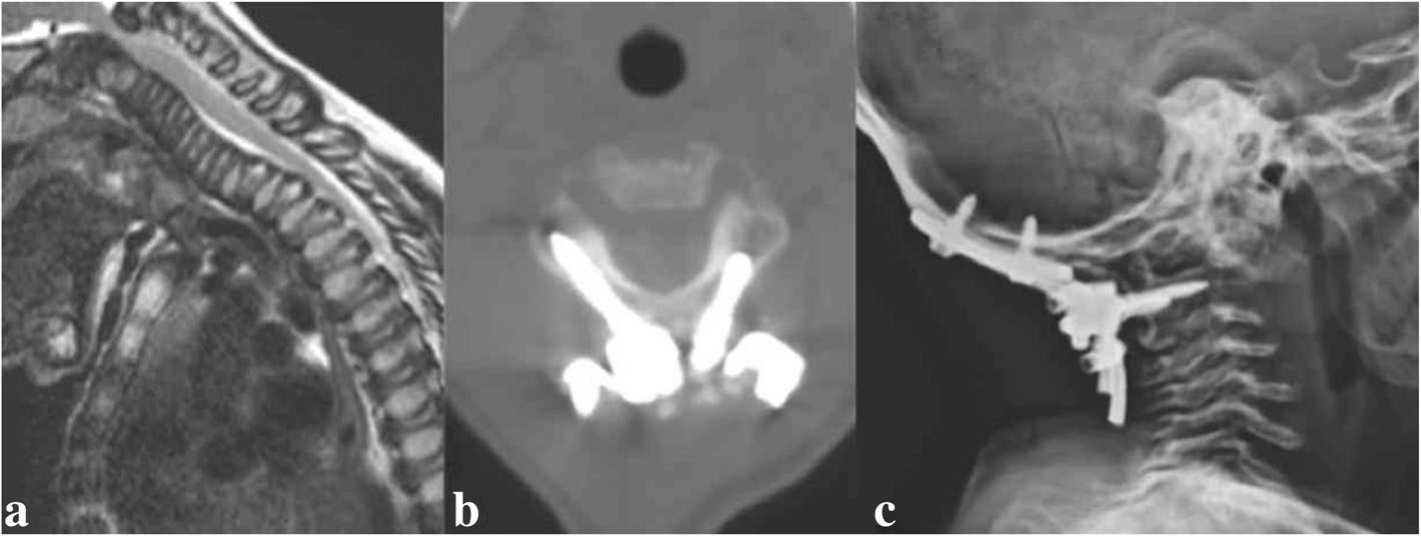
**a** MRI showing severe cord compression during head flexion in MPS IVA, **b** laminar screws and **c** postoperative plain x-rays at 2 years follow-up

A 2-year-old boy with MPS IVA. During routine neuroradiological workup, severe canal stenosis > 50% at the CVJ was observed without signs of myelopathy. Due to the young age of the patient, incomplete development of bony structures at the CVJ, and increased risks of general anesthesia, surgery was schedule at 3 years of age. One year later, follow-up dynamic MRI showed increasing spinal cord compression in flexion (Fig. 2a), although the absence of myelopathy persisted. A preventive CVJ decompression and internal fixation with C2 laminar screws (Fig. 2b) anchored to an occipito-cervical loop augmented with calvarial bone was then performed. Follow-up showed a stable construct (Fig. 2c), without any relevant complication.

### Case 4: Communicating hydrocephalus

**Fig. 3:**
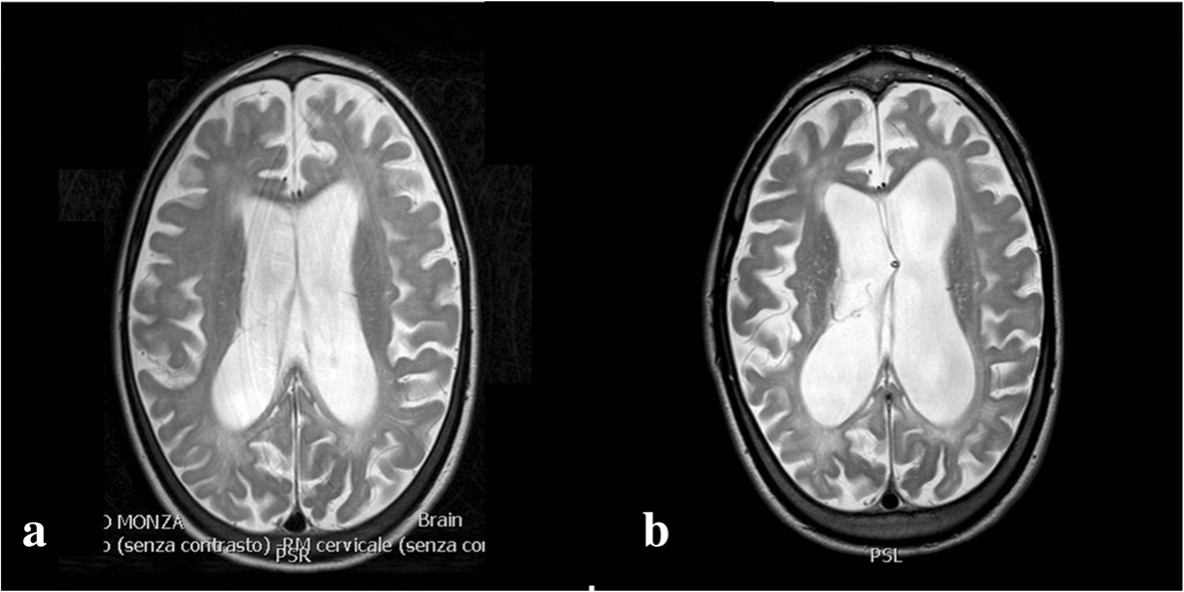
**a** MRI T2-weighted slices showing ventricular dilatation with transependymal reabsorption. **b** A ventriculoperitoneal shunt system was placed, with reduction of ventricular volume and disappearance of periventricular imbibition

A 7-years old MPS II patient. Follow-up MRI including the brain (as in the cases of MPS type I and II) documented a progressive tetraventricular hydrocephalus with transependymal reabsorption (Fig. 3a). Clinically, the patient showed moderate cognitive impairment without specific signs and symptoms of hydrocephalus. Due to the progressive nature of hydrocephalus and radiological evidence of decompensation, a VPS was placed (Fig. 3b), with radiological improvement.

## Conclusion

MPS patients present with a large spectrum of conditions of neurosurgical interest involving both the brain and the spinal cord. Active hydrocephalus is usually slowly progressive and does not routinely require the placement of VPS or an endoscopic intervention, while CVJ pathology is a major feature of these patients for whom surgical management is often recommended and complex. Early recognition is critical to avoid the development of severe complications, especially for CVJ pathology. A multidisciplinary approach involving physicians, neuroradiologists, and neurosurgeons is needed to detect such conditions and to select patients eligible for surgery.

## Abbreviations

ADI: Atlanto-dental interval
CSF: Cerebrospinal fluid
CT: Computed tomography
CVJ: Cranio-vertebral junction
ETV: Endoscopic third ventriculostomy
GAG: Glycosaminoglycan
MPS: Mucopolysaccharidosi(e)s
MRI: Magnetic resonance imaging
SAC: Space Available for the Cord
VPS: Ventriculo-peritoneal shunt

## References

[1] Astarita L, Sibilio M, Andria G. Lysosomal storage disorders: commonalities and differences. In: Parini R, Andria G, editors. Lysosomal storage diseases. Montrouge: Mariani Foundation Paediatric Neurology Series; 2010. p. 3–13.

[2] Montano AM, Tomatsu S, Gottesman GS, Smith M, Orii T (2007). International Morquio a registry: clinical manifestation and natural course of Morquio a disease. J Inherit Metab Dis.

[3] Zafeiriou D, Batzios S (2013). Brain and spinal MR imaging findings in mucopolysaccharidoses: a review. Am J Neuroradiol.

[4] Giussani C, Roux FE, Guerra P, Pirillo D, Grimaldi M, Citerio G, Sganzerla EP (2009). Severely symptomatic craniovertebral junction abnormalities in children: long-term reliability of aggressive management. Pediatr Neurosurg.

[5] Hughes DG, Chadderton RD, Cowie RA, Wraith JE, Jenkins JP (1997). MRI of the brain and craniocervical junction in Morquio's disease. Neuroradiology.

[6] Thorne JA, Javadpour M, Hughes DG, Wraith E, Cowie RA (2001). Craniovertebral abnormalities in type VI mucopolysaccharidosis (Maroteaux-Lamy syndrome). Neurosurgery.

[7] Charrow J, Alden TD, Breathnach CA, Frawley GP, Hendriksz CJ, Link B (2015). Diagnostic evaluation, monitoring, and perioperative management of spinal cord compression in patients with Morquio syndrome. Mol Genet Metab.

[8] Manara R, Priante E, Grimaldi M, Santoro L, Astarita L, Barone R (2011). Brain and spine MRI features of hunter disease: frequency, natural evolution and response to therapy. J Inherit Metab Dis.

[9] Facon D, Ozanne A, Fillard P, Lepeintre JF, Tournoux-Facon C, Ducreux D (2005). MR diffusion tensor imaging and fiber tracking in spinal cord compression. Am J Neuroradiol.

[10] Pueschel SM, Scola FH (1987). Atlantoaxial instability in individuals with Down syndrome: epidemiologic, radiographic, and clinical studies. Pediatrics.

[11] Ransford AO, Crockhard HA, Stevens JM, Modaghegh S (1996). Occipito-atlanto-axial fusion in Morquio-Brailsford syndrome. A ten-year experience. J Bone Joint Surg Br.

[12] Mut M, Cila A, Varli K, Akalan N (2005). Multilevel myelopathy in Maroteaux–Lamy syndrome and review of the literature. Clin Neurol Neurosurg.

[13] Sganzerla EP, Giussani C, Grimaldi M, Parini R, Ingelmo P, Trezza A, Visocchi M (2014). Craniovertebral junction pathological features and their management in the mucopolysaccharidoses. Adv Tech Stand Neurosurg.

[14] Ahmed R, Traynelis VC, Menezes AH (2008). Fusions at the craniovertebral junction. Childs Nerv Syst.

[15] Dickerman RD, Colle KO, Bruno CA, Schneider SJ (2004). Craniovertebral instability with spinal cord compression in a 17-month-old boy with sly syndrome (mucopolysaccharidosis type VII): a surgical dilemma. Spine.

[16] Anderson RC, Ragel BT, Mocco J, Bohman LE, Brockmeyer DL (2007). Selection of a rigid internal fixation construct for stabilization at the craniovertebral junction in pediatric patients. J Neurosurg.

[17] Couture D, Avery N, Brockmeyer DL (2010). Occipitocervical instrumentation in the pediatric population using a custom loop construct: initial results and long-term follow-up experience. J Neurosurg Pediatr.

[18] Visocchi M, Di Rocco F, Meglio M (2003). Craniocervical junction instability: instrumentation and fusion with titanium rods and sublaminar wires. Effectiveness and failures in personal experience. Acta Neurochir.

[19] Visocchi M, Della Pepa GM, Doglietto F, Esposito G, La Rocca G, Massimi L (2011). Video-assisted microsurgical transoral approach to the craniovertebral junction: personal experience in childhood. Childs Nerv Syst.

[20] Visocchi M, Pietrini D, Tufo T, Fernandez E, Di Rocco C (2009). Preoperative irreducible C1-C2 dislocations: intraoperative reduction and posterior fixation. The always posterior strategy. Acta Neurochir.

[21] Matheus MG, Castillo M, Smith JK, Armao D, Towle D, Muenzer J (2004). Brain MRI findings in patients with mucopolysaccharidosis types I and II and mild clinical presentation. Neuroradiology.

[22] Lee C, Dineen TE, Brack M, Kirsch JE, Runge VM (1993). The mucopolysaccharidoses: characterization by cranial MR imaging. Am J Neuroradiol.

[23] Yin LK, Zheng JJ, Zhao L, Hao XZ, Zhang XX, Tian JQ (2017). Reversed aqueductal cerebrospinal fluid net flow in idiopatich normal pressire hydrocephalus. Acta Neurol Scand.

[24] Beck M, Cole G (1984). Disc oedema in association with Hunter’s syndrome: ocular histopathological findings. Br J Ophthalmol.

[25] Neto ÂR, Holanda GB, Farias MC, Santos da Costa G, Pereira HS (2013). Hydrocephalus in mucopolysaccharidosis type VI successfully treated with endoscopic third ventriculostomy. J Neurosurg Pediatr.

[26] Aliabadi H, Reynolds R, Powers CJ, Grant G, Fuchs H, Kurtzberg J (2010). Clinical outcome of cerebrospinal fluid shunting for communicating hydrocephalus in mucopolysaccharidoses I, II, and III: a retrospective analysis of 13 patients. Neurosurgery.

